# Description of antibiotic stewardship expertise and activities among US public health departments, 2022

**DOI:** 10.1017/ash.2023.211

**Published:** 2023-09-29

**Authors:** Destani Bizune, Angelina Luciano, Melinda Neuhauser, Lauri Hicks, Sarah Kabbani

## Abstract

**Background:** In 2021, the CDC awarded >$100 million to 62 state, local, and territorial health departments (SLTHDs) to expand antibiotic stewardship expertise and implement antibiotic stewardship activities in different healthcare settings. Our objective was to describe SLTHD antibiotic stewardship personnel and activities to characterize the impact of the funding. **Methods:** SLTHDs submitted performance measures, including quantitative and qualitative responses, describing personnel supporting antibiotic stewardship activities, types of activities, and healthcare facilities and professionals engaged from January through June 2022. A quantitative analysis of performance measures and qualitative thematic analysis of select narrative responses are reported. **Results:** Most SLTHDs (58 of 62, 94%) submitted performance measures. Among them, 37 (64%) reported identifying an antibiotic stewardship leader or coleader; most were pharmacists (57%) or physicians (38%) with infectious diseases training (68%) (Table 1). Of the remaining STLHDs, 20 reported barriers to identifying a leader or coleader, including hiring process delays and programmatic barriers (Table 2). SLTHDs reported 254 antibiotic stewardship activities; most reported activities involving multiple activity types (44%). Education and communication (eg, providing stewardship expertise) was the most common single activity (30%), followed by antibiotic use tracking and reporting (13%), assessment of antibiotic stewardship implementation (8%), and action and implementation (eg, audit and feedback letters) (4%). The highest number of activities were implemented in multiple healthcare settings (35%), followed by acute care (21%), outpatient (18%), long-term care (17%), and other (9%) (Fig. 1). SLTHDs reported engaging 4,970 healthcare facilities and 15,194 healthcare professionals in antibiotic stewardship activities across healthcare settings, to date, as part of this funding opportunity (Fig. 2). **Conclusions:** Antibiotic stewardship funding to SLTHDs allowed for increases in capacity and expanded outreach to implement a variety of antibiotic stewardship activities across multiple healthcare settings. Sustaining STLHD antibiotic stewardship activities can help increase engagement and coordination with healthcare facilities, healthcare professionals, and other partners to optimize antibiotic prescribing and patient safety.

**Figure:**
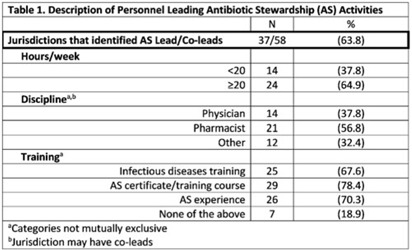

**Figure:**
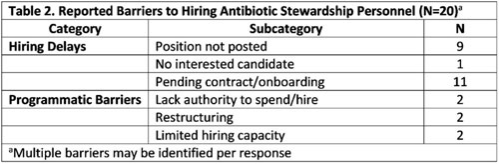

**Figure:**
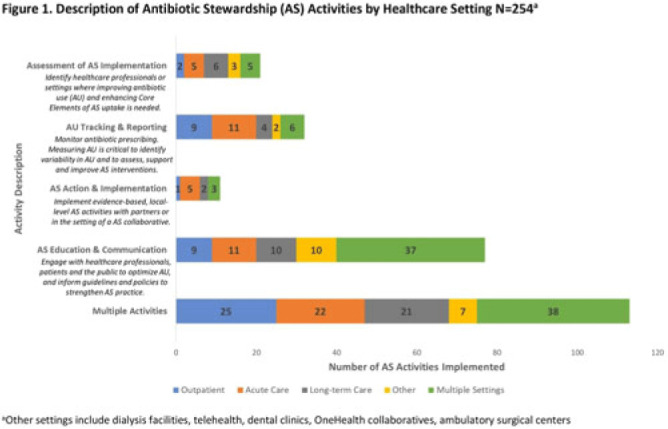

**Figure:**
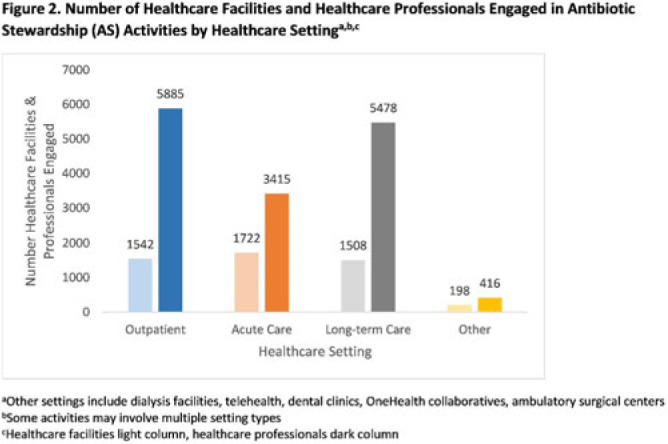

**Disclosure:** None

